# Machine-Learning Predictions of Photoluminescence in Molecules Exhibiting Thermally Activated Delayed Fluorescence with Implicit Experimental Validation

**DOI:** 10.1021/acs.jcim.6c00425

**Published:** 2026-05-13

**Authors:** Dingyun Huang, Jacqueline M. Cole

**Affiliations:** Ray Dolby Centre, Cavendish Laboratory, Department of Physics, 2152 University of Cambridge, J. J. Thomson Avenue, Cambridge CB3 0US. U.K

## Abstract

The application of machine learning to materials discovery is often constrained by the availability of large-scale, experimentally verified materials databases. This study presents an automatic, end-to-end framework that bridges this gap by training machine-learning predictors for materials properties on experimental data mined directly from the literature. We apply this framework to predict the photoluminescence (PL) wavelengths of thermally activated delayed fluorescence molecules. By integrating “chemistry-aware” natural language processing with automated chemical structure resolution, a dataset of 643 experimentally measured PL wavelengths was afforded. This experimentally grounded data were used to train a heterogeneous graph neural network and a ridge-regression model; both achieved mean absolute errors below 0.13 eV in less than 3 min on a personal laptop, effectively capturing complex structure–property relationships without manual feature engineering. These results demonstrate that our framework provides a fast, scalable, and generalizable pathway to generate experimentally grounded models for property predictions in organic optoelectronics.

## Graphical abstract

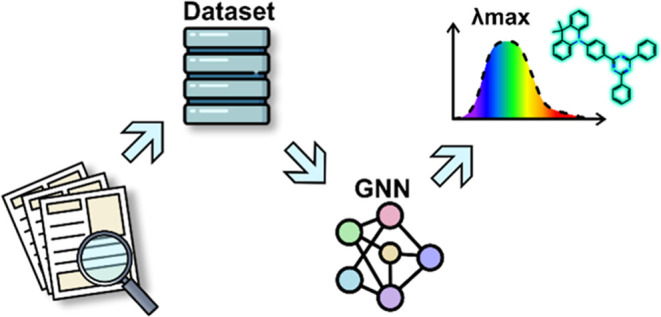

## Introduction

Over the past decade, data-driven and machine-learning methods have emerged as a promising approach for accelerating materials characterization and discovery.^1–12^ Yet, the materials databases required for realizing these approaches are often unavailable owing to excessive costs of ab initio calculation involved in simulated datasets or the limited availability of large-scale crowd-sourced experimental databases. Furthermore, simulated databases are sometimes criticized for oversimplification and numerical errors,^13,14^ potentially diverging from experimental realities. Therefore, demand has risen for machine-learning models that are trained on and validated by experimental data.

Thermally activated delayed fluorescence (TADF) molecules are strong candidates for next-generation commercial organic light-emitting diodes. They have a theoretical internal quantum efficiency of 100% by harvesting both singlet and triplet excitons while offering improved processability and cost-efficiency.^15–21^ Since TADF molecules often contain many non-hydrogen atoms, generating simulated databases by ab initio electronic-structure calculations can be a problematic option due to the exponentially scaling cost of computation.^22–26^ Consequently, there are few comprehensive databases about TADF molecules, obstructing the application of data-driven methods in the field. Similar situations are also experienced by the general organic electronics research community. Determining photoluminescence (PL) wavelengths, a key functional attribute, is particularly costly. It involves the optimization of ground-state and excited-state geometries, which poses a bigger challenge for ab initio simulations. A fast and accurate predictor for PL wavelengths of TADF molecules could significantly improve efficiency in both experimental and computational studies of these materials.

Fortunately, a vast quantity of experimental materials data exist within the scientific literature in unstructured formats, and recent efforts have shown that systematic databases of these data can be automatically extracted using natural language processing techniques or large language models.^27–35^ Such databases can enable machine learning (ML) models grounded in experimental measurements, implicitly incorporating real-world conditions, while avoiding the prohibitive computational costs incurred by simulating databases. Nevertheless, the extraction of molecular-structure representations from text remains challenging because this information is usually presented in papers as chemical schematic diagrams instead of text. Consequently, many data-driven prediction models tend to use only chemical formulas as the structure input.^36,37^ This heavily limits the level of structural input about TADF molecules to these data-driven predictors.

To improve upon this overall situation, this study introduces an end-to-end framework that automatically mines experimental PL wavelengths of TADF molecules and the solvents used in the measurements from the literature and trains ML models on these data. The text-mining workflow computes the simplified molecular input line entry system (SMILES) string from the IUPAC name for a molecule and property record via abbreviation detection and coreference resolution.^29^ The SMILES strings serve as the structural information input for the machine-learning models. As a result, a database, TadfPL, of 643 PL wavelength records was obtained. Subsequently, we trained a heterogeneous graph neural network (HGNN) model and a ridge-regression model independently. Both experimentally grounded models obtained mean absolute errors (MAEs) below 0.13 eV. The presented framework can be easily generalized to other photophysical properties of TADF molecules, such as PL quantum yield and singlet–triplet energy splitting, and to other organic electronic materials. A schematic of the proposed framework is shown in Figure 1a.

**Figure 1:**
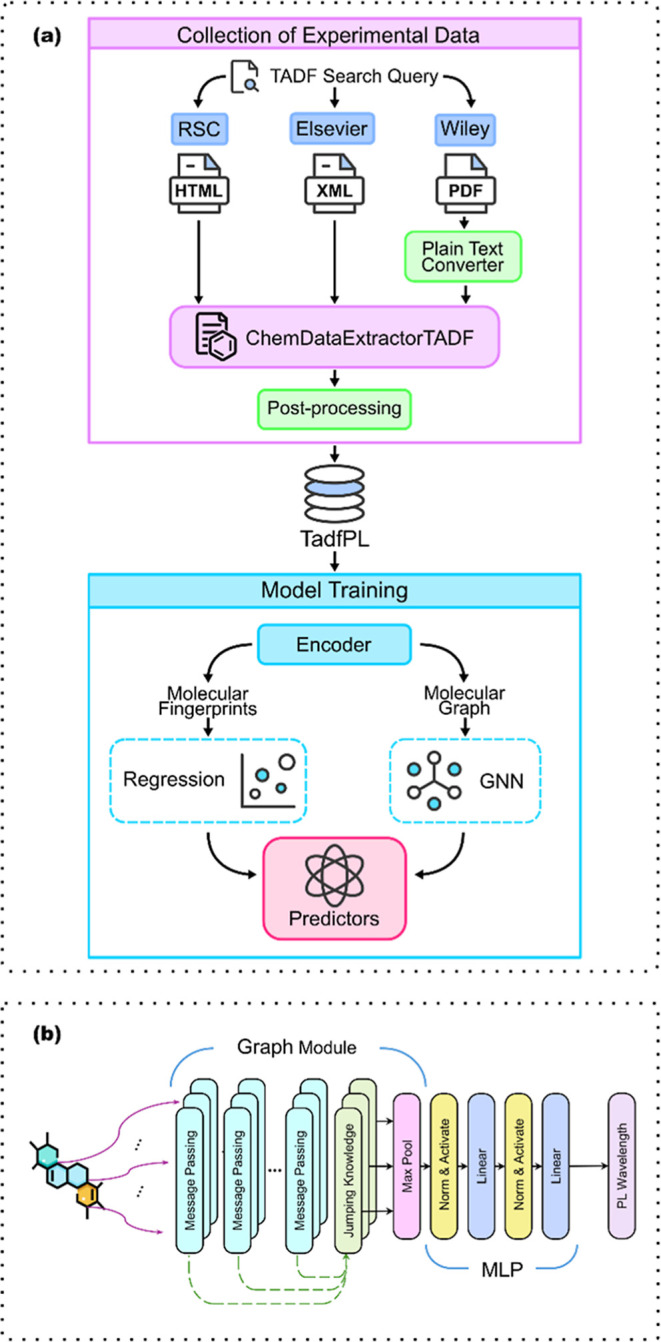
(a) Two key stages of the proposed PL prediction framework: experimental data collection, and model training. The data collection was achieved by mining text in publications from the RSC, Elsevier, and Wiley using ChemDataExtractorTADF. The output dataset, TadfPL, was used to train a HGNN model on molecular graphs and a linear-regression model on molecular fingerprints. (b) Architecture of the HGNN model used in this work.

## Methods

First, a bespoke dataset, TadfPL, of experimental PL wavelengths measured in solution and their associated molecular SMILES strings were automatically extracted from the literature using ChemDataExtractor and other text-mining techniques. Second, two machine-learning methods were explored on the extracted dataset for predicting the peak PL wavelength from the SMILES string of a TADF molecule. One model was developed by using a conventional method of molecular fingerprinting and ridge-regression. Meanwhile, a HGNN was adopted for the other model whose architecture is illustrated in Figure 1b. The HGNN architecture used in this study was derived from GraphSAGE; modifications to the original GraphSAGE model and the hyperparameters used are given in Supporting Information S4.

The TadfPL dataset was collected by mining text about organic TADF molecules from the Royal Society of Chemistry (RSC), Elsevier, and Wiley publishers, using the pipeline proposed by Huang and Cole.^29^ Overall, the data-extraction process involves three stages: (1) searching and downloading relevant publications from each publisher, (2) mining text for properties and chemical names, and (3) data postprocessing and cleaning. The following modifications were implemented to boost extraction precision and enhance code efficiency.

First of all, research articles about organic TADF molecules were retrieved from the three publishers by querying their databases with search phrases and filters that were carefully designed to avoid irrelevant papers. Papers from the RSC and Elsevier are in hypertext markup language (HTML) and extensive markup language (XML) files, respectively, which are machine-readable and ready for mining. Since portable document format (PDF) is a fixed-layout presentation format, such files are difficult to text-mine because they do not contain structured text. Hence, a new text-extraction algorithm was developed for mining PDF files of Wiley journals, using MinerU and ChemDataExtractor.^38^ In this work, only those PL wavelength records that have been measured in solution were extracted to ensure a data consistency for modeling. Accordingly, we extracted data only from tables in the documents because table headers and footnotes allow an easy separation of PL wavelengths that have been measured in solution from electroluminescence (EL) and other PL measurements in films and devices. During postprocessing, the data format was standardized, and SMILES strings were computed for valid IUPAC names. The dataset was then cleaned and corrected for four major types of extraction errors identified for our data-extraction pipeline, listed in Table S1. A more detailed description of the entire data-mining workflow is given in Supporting Information S1.

## Results and Discussion

The overall data-mining workflow yielded a dataset, TadfPL, consisting of 643 unique data records of PL maxima and the solvents used. There are 361 ground-truth records in the dataset as they have been verified in the data-cleaning stage of the data postprocessing pipeline. The TadfPL dataset is split into training, validation, and test sets in a stratified fashion so that the PL wavelength distributions of the three subsets are similar, as shown in Figure 2a. The violin plots display the smoothed PL distributions of the three data subsets, and the box plots label the medians and interquartile ranges (IQR). When splitting this dataset, we also ensured that no duplicate molecules span across the three splits and that the validation and test sets contain only ground-truth data records. These approaches were applied to prevent information leakage to the training stage of the property prediction process and to achieve robust performance estimations.

**Figure 2:**
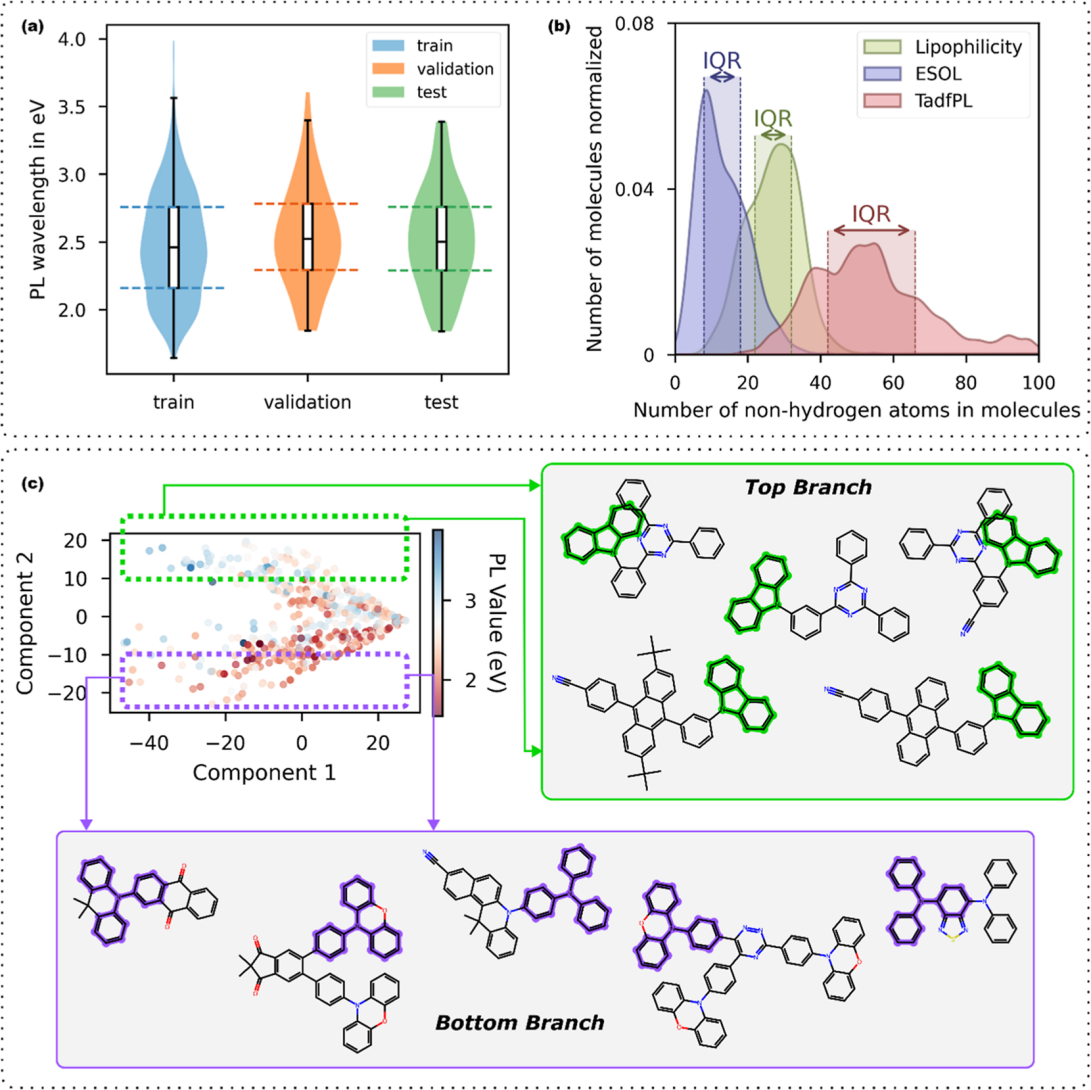
(a) The training, validation, and test sets are split from TadfPL in a stratified manner so that the distributions of their PL values are similar, as demonstrated by the violin plots. The solid bar at the midpoint of each embedded box plot represents the median of each distribution, while two dotted lines at the opposing ends of each box plot represent the upper and lower quartiles, such that the full width of each box plot denotes the interquartile range (IQR). (b) Normalized and smoothed distributions of non-hydrogen atom numbers in molecules from TadfPL, as well as the two benchmarks, ESOL and Lipophilicity, where the IQR of each distribution is shaded and labeled. Molecules from TadfPL are on average significantly larger than those in the other two benchmark datasets; the spread in molecular size of TadfPL is also wider. (c) Principal component analysis (PCA) plot of molecular fingerprints for TadfPL entries. Two branches above and below the axis of Component 1 emerge in the plot, which can be attributed to whether the molecule contains carbazoles or triphenylamines as a substructure. Five molecules from each branch were drawn, and the matching substructures are highlighted.

Figure 2b illustrates the distribution of molecules from the TadfPL dataset in terms of their number of non-hydrogen atoms. Distributions from two common organic molecule benchmark datasets, ESOL and Lipophilicity, are also included for comparison.^39,40^ The IQR of the distribution of TadfPL lies entirely to the right of IQRs of ESOL and Lipophilicity. This evidences that molecules from TadfPL tend to be significantly larger than those from the two other benchmarks; its spread in molecular size is also much wider. Additional descriptive statistics of the three distributions are given in Table S2. These features of TadfPL pose challenges to common machine-learning and deep-learning methods for organic molecules as those methods have primarily been developed and evaluated on datasets of smaller molecules. The scatter plot resulting from a PCA on RDKit^41^ fingerprint vectors of molecules in TadfPL (Figure 2c) signifies two main branches, separated by the first principal component axis (PC1). Five molecules were randomly sampled with PC2 > 10 and another five with PC2 < –10, whose schematics are displayed. One can observe that the molecules with PC2 > 10 all contain the carbazole molecular substructure, while those with PC2 < –10 all contain the triphenylamine molecular fragment instead. Both molecular moieties are common in TADF molecular design, and the branching feature in the PCA plot can be approximately attributed to the distinction between containing one or the other substructures. As carbazole moieties often function as electron donors with low highest occupied molecular orbital energies in donor–acceptor type TADF molecules, it stands to reason that the top branch in the scatter plot, which pertains to carbazole-containing molecules, has overall higher photoluminescence energies in TadfPL. However, a similar argument does not apply to the bottom branch as the triphenylamine fingerprint appears in both the donor and acceptor moieties of the examples displayed.

The RDKit^41^ fingerprint was adopted for its widespread use to encode molecules for training the ridge-regression model, where each fingerprint represents a subgraph of the molecular skeleton. These methods are subject to hash collisions, where multiple molecular fingerprints are mapped to the same element of a feature vector. The rate of such collisions rises rapidly with the number of atoms in molecules (Figure S1). The combined effects of feature-vector length and subgraph sizes were investigated with regard to these hash collisions (Supporting Information S3 and Figure S2). This revealed a trend from which we extrapolated that fingerprints produced with minPath = 4, maxPath = 10, and fpSize = 65,536 were found to afford the best ridge-regression performance. The model was evaluated on the out-of-sample test set, and an MAE of 0.129 eV was obtained.

Meanwhile, a hyperparameter sweep was performed to select the optimal configuration for the proposed HGNN model, based on the performance on the validation set. The number of parameters of the optimal configuration is 55,369, less than 65,537 in the ridge-regression model. Details of the sweep and the optimal HGNN configuration are given in the Supporting Information S4. Subsequently, five randomly initialized models were trained on the combined training and validation set using the determined optimal configuration and evaluated on the out-of-sample test set. An average MAE of 0.119 ± 0.002 eV was obtained, outperforming the method of ridge-regression on molecular fingerprints.

Table 1 compares the performance of the ridge-regression model and the HGNN developed in this study with two baseline models: the original GraphSAGE architecture and a gradient-boosted decision tree model. To ensure a fair comparison, the hyperparameters of both baseline models were optimized independently, and the resulting optimal configurations are provided in Supporting Information S5. Notably, the HGNN contains only one-sixth as many parameters as the original homogeneous GraphSAGE model while achieving comparable predictive performance. Such a smaller model size also considerably reduces the amount of data required for the training. For additional context, Table 1 also includes a smaller GraphSAGE model with a parameter count similar to that of the HGNN; however, its performance is slightly worse than that of the ridge-regression model. The configuration of this smaller GraphSAGE model is also given in Supporting Information S5.

**Table 1:** Number of Parameters, MAEs, and ***R*** ^2^s of the Five Methods Investigated in This Study

	no. of parameters	MAE (eV)	***R*** ^2^
ridge-regression	65,537	0.129	0.79
GBDT	-	0.155	0.70
GraphSAGE	333,721	0.120	0.80
HGNN	55,369	0.119	0.80
GraphSAGE (small)	51,833	0.133	0.75

Predicted PL wavelengths on training and test sets of the ridge-regression model and the HGNN model are plotted against the labeled values in Figure 3a,c. The error distributions of both models are given in Figure 3e. HGNN prediction errors are centered more narrowly around zero than those from the ridge-regression model. Residual plots of the HGNN and ridge-regression model predictions are plotted against true PL values in Figure 3d,f. Both scatter points illustrate weak negative correlation, indicating negative bias, which means that the models underestimate PL values for molecules with large PL signatures and vice versa. Wald tests yielded *p*-values of 8 × 10^–5^ and 3 × 10^–7^ for the HGNN and ridge-regression model, respectively, suggesting strong evidence for nonzero bias. As our training and test datasets are small, this bias could be reduced significantly with a larger dataset. Three well-known TADF molecules, PXZ-TRZ, 4CzIPN, and DMAC-TRZ, and their prediction errors are also listed in Figure 3b; all are predicted with less than 0.1 eV error from either model. Furthermore, the process of calculating fingerprints and fitting a ridge-regression model took only 1.5 min, while training of a HGNN model comparably took around 2.5 min on a laptop with an RTX 3050 GPU; thus, the framework is readily executable on personal computers.

**Figure 3:**
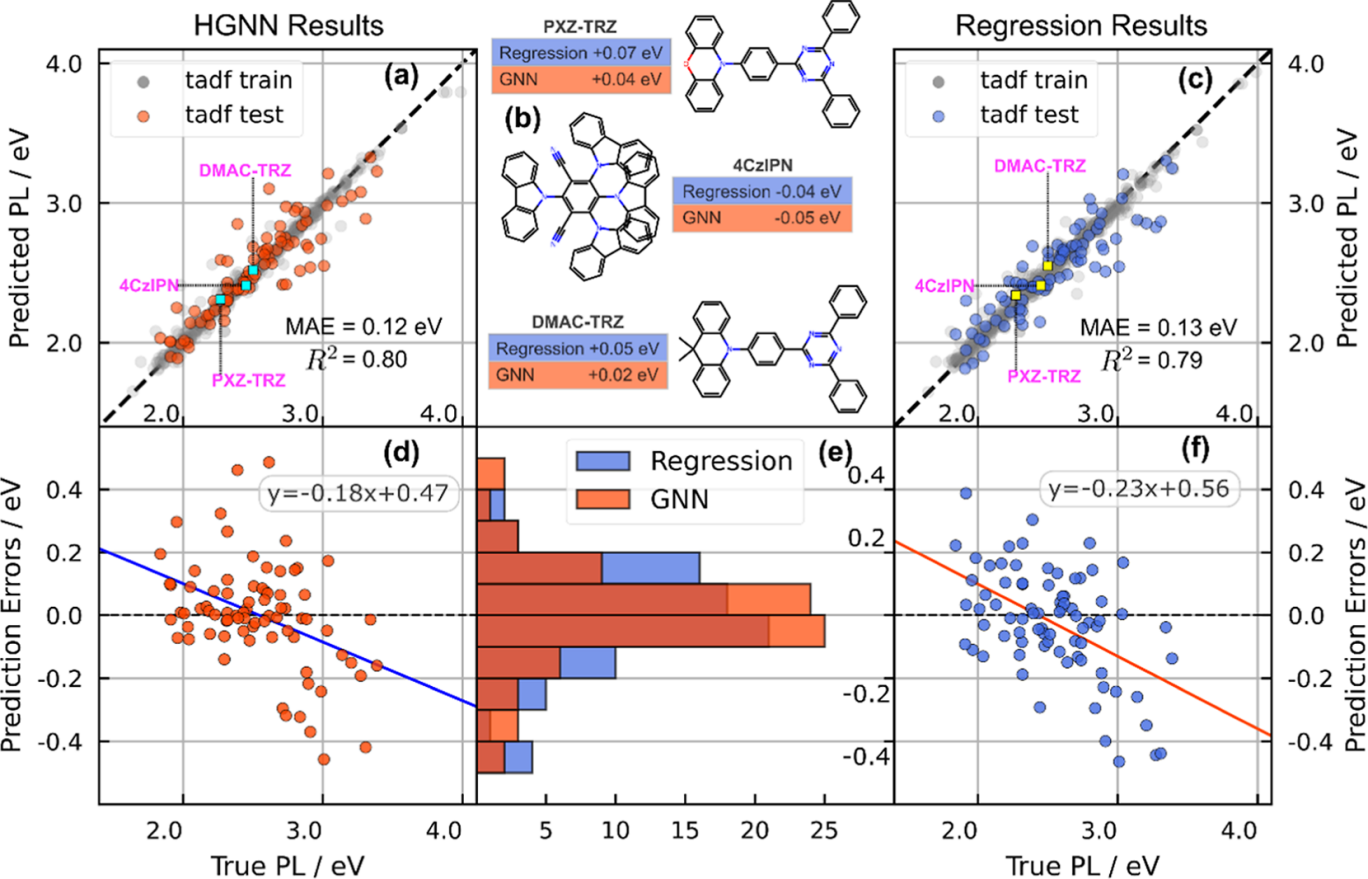
(a,c) Predicted PL against true PL for train and test set from the HGNN and regression model, respectively, and their MAEs and *R* ^2^ are labeled. (d,f) Prediction residuals against the true PL values for each model, where a negative bias can be observed for both. (e) The residual distributions for both models; a narrower spread around zero is seen for the HGNN model. (b) Schematics of three common TADF emitters and prediction errors on them from both models; all errors are less than 0.1 eV.

The PL wavelengths of TADF molecules in solution are highly sensitive to solvent polarity, particularly for donor–acceptor designs. Thus, it is, in principle, advantageous to train an HGNN model of the same architecture with solvent information included as an additional input feature. However, 70% of the records in TadfPL was measured in toluene, while the next two most common solvents, tetrahydrofuran and dichloromethane, account for 12% and 11% of the dataset, respectively. This pronounced data imbalance reflects the prevailing solvent choices within the TADF research community for PL measurements, thereby introducing substantial bias into the solvent features of all associated ML models. To assess its impact, an additional HGNN model was trained with solvent identity appended as a one-hot encoded vector, yielding an MAE of 0.131 eV. Furthermore, training an HGNN model exclusively on those data in TadfPL that have been measured in toluene resulted in an MAE of 0.129 eV. Although the performance of these models is slightly inferior to the best result reported in this work, the incorporation of solvent information into PL predictions may enhance the robustness of the ML model when trained on a dataset with a more balanced solvent distribution.

## Conclusions

Overall, a general framework has been presented for modeling the photoluminescence wavelengths of TADF molecules using a data-driven approach with implicit experimental validation. The framework begins with mining text from the scientific literature using ChemDataExtractorTADF, yielding a TadfPL dataset that constitutes 643 structure–property pairs. A new PDF mining algorithm was designed for extracting data from Wiley Journal PDF as part of the process. A ridge-regression model with molecular fingerprints and a HGNN model were trained on these mined experimental data to predict PL wavelengths using only SMILES strings as input; both data-driven predictors achieved small MAE ≤0.13 eV. Despite the small dataset, these predictors achieved strong performance, and it is expected that their performance and robustness can be further improved using larger datasets of experimental PL values. The use of experimental training data is unusual, but highly beneficial, because it allows the ML models to be implicitly verified by the experiment. Finally, the framework can be readily generalized to predict more properties of TADF molecules and other organic molecular materials, thus accelerating materials screening and discoveries in these domains.

## Supplementary Material





## Data Availability

TADF photoluminescence prediction models and code are available in the following GitHub repository: Dingyun-Huang/tadf-photoluminescence-prediction.

## Notes

Text-mining workflow used to collect the TadfPL dataset; statistics of the TadfPL, ESOL, and Lipophilicity datasets; hash-collision effects in molecular fingerprints; and HGNN model configuration and training (PDF)
TadfPL dataset; list of DOIs of articles that have been text-mined in this study; and TadfPL dataset at each stage of postprocessing (ZIP)

## References

[1] Jung S. G., Jung G., Cole J. M. (2024). Automatic Prediction of Peak Optical
Absorption Wavelengths in Molecules Using Convolutional Neural Networks. J. Chem. Inf. Model..

[2] Harris S. B., Gemperline P. T., Rouleau C. M., Vasudevan R. K., Comes R. B. (2025). Deep Learning with Reflection High-Energy Electron
Diffraction Images to Predict Cation Ratio in Sr2xTi2­(1–x)­O3
Thin Films. Nano Lett..

[3] Yang H., Hu C., Zhou Y., Liu X., Shi Y., Li J., Li G., Chen Z., Chen S., Zeni C., Horton M., Pinsler R., Fowler A., Zügner D., Xie T., Smith J., Sun L., Wang Q., Kong L., Liu C., Hao H., Lu Z. (2024). MatterSim: A Deep Learning Atomistic
Model Across Elements, Temperatures and Pressures. arXiv.

[4] Zeni C., Pinsler R., Zügner D., Fowler A., Horton M., Fu X., Wang Z., Shysheya A., Crabbé J., Ueda S., Sordillo R., Sun L., Smith J., Nguyen B., Schulz H., Lewis S., Huang C.-W., Lu Z., Zhou Y., Yang H., Hao H., Li J., Yang C., Li W., Tomioka R., Xie T. (2025). A Generative
Model for Inorganic Materials Design. Nature.

[5] Merchant A., Batzner S., Schoenholz S. S., Aykol M., Cheon G., Cubuk E. D. (2023). Scaling Deep Learning
for Materials Discovery. Nature.

[6] Cole J. M., Low K. S., Ozoe H., Stathi P., Kitamura C., Kurata H., Rudolf P., Kawase T. (2014). Data Mining with Molecular
Design Rules Identifies New Class of Dyes for Dye-Sensitised Solar
Cells. Phys. Chem. Chem. Phys..

[7] Gómez-Bombarelli R., Aguilera-Iparraguirre J., Hirzel T. D., Duvenaud D., Maclaurin D., Blood-Forsythe M. A., Chae H. S., Einzinger M., Ha D.-G., Wu T., Markopoulos G., Jeon S., Kang H., Miyazaki H., Numata M., Kim S., Huang W., Hong S. I., Baldo M., Adams R. P., Aspuru-Guzik A. (2016). Design of Efficient Molecular Organic Light-Emitting
Diodes by a High-Throughput Virtual Screening and Experimental Approach. Nat. Mater..

[8] Jain A., Ong S. P., Hautier G., Chen W., Richards W. D., Dacek S., Cholia S., Gunter D., Skinner D., Ceder G., Persson K. A. (2013). Commentary: The Materials Project:
A Materials Genome Approach to Accelerating Materials Innovation. APL Mater..

[9] Ramakrishnan R., Dral P. O., Rupp M., von Lilienfeld O. A. (2014). Quantum
Chemistry Structures and Properties of 134 Kilo Molecules. Sci. Data.

[10] Groom C. R., Bruno I. J., Lightfoot M. P., Ward S. C. (2016). The Cambridge Structural
Database. Acta Cryst. B.

[11] Ju C.-W., Bai H., Li B., Liu R. (2021). Machine Learning Enables Highly Accurate
Predictions of Photophysical Properties of Organic Fluorescent Materials:
Emission Wavelengths and Quantum Yields. J.
Chem. Inf. Model..

[12] Zou Z., Zhang Y., Liang L., Wei M., Leng J., Jiang J., Luo Y., Hu W. (2023). A Deep Learning Model
for Predicting Selected Organic Molecular Spectra. Nat. Comput. Sci..

[13] Leeman J., Liu Y., Stiles J., Lee S. B., Bhatt P., Schoop L. M., Palgrave R. G. (2024). Challenges in High-Throughput
Inorganic Materials Prediction
and Autonomous Synthesis. PRX Energy.

[14] Kuryla D., Berger F., Csányi G., Michaelides A. (2025). How Accurate
Are DFT Forces? Unexpectedly Large Uncertainties in Molecular Datasets. arXiv.

[15] Chen J.-X., Wang K., Zheng C.-J., Zhang M., Shi Y.-Z., Tao S.-L., Lin H., Liu W., Tao W.-W., Ou X.-M., Zhang X.-H. (2018). Red Organic Light-Emitting
Diode
with External Quantum Efficiency beyond 20% Based on a Novel Thermally
Activated Delayed Fluorescence Emitter. Adv.
Sci..

[16] Uoyama H., Goushi K., Shizu K., Nomura H., Adachi C. (2012). Highly Efficient
Organic Light-Emitting Diodes from Delayed Fluorescence. Nature.

[17] Adachi C., Baldo M. A., Thompson M. E., Forrest S. R. (2001). Nearly 100% Internal
Phosphorescence Efficiency in an Organic Light-Emitting Device. J. Appl. Phys..

[18] Zhang K., Yang F., Zhang Y., Ma Y., Fan J., Fan J., Wang C.-K., Lin L. (2021). Highly Efficient Near-Infrared Thermally
Activated Delayed Fluorescence Molecules via Acceptor Tuning: Theoretical
Molecular Design and Experimental Verification. J. Phys. Chem. Lett..

[19] Patil V. V., Lim J., Cho S. M., Lee J. Y. (2024). Highly
Efficient C/N-Fused Architecture
for Narrowband Deep-Blue Thermally Activated Delayed Fluorescence. Adv. Opt. Mater..

[20] Huang Z., Xie H., Miao J., Wei Y., Zou Y., Hua T., Cao X., Yang C. (2023). Charge Transfer Excited State Promoted Multiple Resonance
Delayed Fluorescence Emitter for High-Performance Narrowband Electroluminescence. J. Am. Chem. Soc..

[21] Dos
Santos J. M., Hall D., Basumatary B., Bryden M., Chen D., Choudhary P., Comerford T., Crovini E., Danos A., De J., Diesing S., Fatahi M., Griffin M., Gupta A. K., Hafeez H., Hämmerling L., Hanover E., Haug J., Heil T., Karthik D., Kumar S., Lee O., Li H., Lucas F., Mackenzie C. F. R., Mariko A., Matulaitis T., Millward F., Olivier Y., Qi Q., Samuel I. D. W., Sharma N., Si C., Spierling L., Sudhakar P., Sun D., Tankelevičiu̅tė E., Duarte Tonet M., Wang J., Wang T., Wu S., Xu Y., Zhang L., Zysman-Colman E. (2024). The Golden Age of Thermally Activated
Delayed Fluorescence Materials: Design and Exploitation. Chem. Rev..

[22] Froitzheim T., Kunze L., Grimme S., Herbert J. M., Mewes J.-M. (2024). Benchmarking
Charge-Transfer Excited States in TADF Emitters: ΔDFT Outperforms
TD-DFT for Emission Energies. J. Phys. Chem.
A.

[23] Jacquemin, D. ; Adamo, C. Computational Molecular Electronic Spectroscopy with TD-DFT. In Density-Functional Methods for Excited States; Ferré, N. , Filatov, M. , Huix-Rotllant, M. , Eds.; Springer International Publishing: Cham, 2016; pp 347–375. 10.1007/128_2015_638. 26003564

[24] Younker J. M., Dobbs K. D. (2013). Correlating Experimental Photophysical
Properties of
Iridium­(III) Complexes to Spin–Orbit Coupled TDDFT Predictions. J. Phys. Chem. C.

[25] Ahmad S. A., Eng J., Penfold T. J. (2022). Rapid Predictions of the Colour Purity of Luminescent
Organic Molecules. J. Mater. Chem. C.

[26] Peñuelas-Gámez C. A., Luque-Román M., Báez-Castro A., Delgado-Montiel T., Soto-Acosta S., Soto-Rojo R., Ceballos-Mendivil L., Glossman-Mitnik D., Baldenebro-López J. (2026). Triphenylamine vs Triphenylphosphine
Donors in D-A and D-π-A Frameworks: A DFT/TD-DFT Roadmap for
DSSCs and OLEDs. J. Fluoresc..

[27] Court C. J., Cole J. M. (2018). Auto-Generated Materials Database
of Curie and Néel
Temperatures via Semi-Supervised Relationship Extraction. Sci. Data.

[28] Huang S., Cole J. M. (2020). A Database of Battery
Materials Auto-Generated Using
ChemDataExtractor. Sci. Data.

[29] Huang D., Cole J. M. (2024). A Database of Thermally Activated
Delayed Fluorescent
Molecules Auto-Generated from Scientific Literature with ChemDataExtractor. Sci. Data.

[30] Dong Q., Cole J. M. (2022). Auto-Generated Database
of Semiconductor Band Gaps
Using ChemDataExtractor. Sci. Data.

[31] Zheng Z., Zhang O., Borgs C., Chayes J. T., Yaghi O. M. (2023). ChatGPT
Chemistry Assistant for Text Mining and the Prediction of MOF Synthesis. J. Am. Chem. Soc..

[32] Dai D., Zhang G., Wei X., Lin Y., Dai M., Peng J., Song N., Tang Z., Li S., Liu J., Xu Y., Che R., Zhang H. (2025). A GPT-Assisted Iterative
Method for Extracting Domain Knowledge from a Large Volume of Literature
of Electromagnetic Wave Absorbing Materials with Limited Manually
Annotated Data. Comput. Mater. Sci..

[33] Zhang X., Zhou Z., Ming C., Sun Y.-Y. (2023). GPT-Assisted Learning
of Structure–Property Relationships by Graph Neural Networks:
Application to Rare-Earth-Doped Phosphors. J.
Phys. Chem. Lett..

[34] Huang D., Cole J. M. (2025). Cost-Efficient Domain-Adaptive Pretraining
of Language
Models for Optoelectronics Applications. J.
Chem. Inf. Model..

[35] Isazawa T., Cole J. M. (2024). How Beneficial Is Pretraining on
a Narrow Domain-Specific
Corpus for Information Extraction about Photocatalytic Water Splitting?. J. Chem. Inf. Model..

[36] Jung S. G., Jung G., Cole J. M. (2024). Machine-Learning
Predictions of Critical
Temperatures from Chemical Compositions of Superconductors. J. Chem. Inf. Model..

[37] Court C. J., Cole J. M. (2020). Magnetic and Superconducting Phase
Diagrams and Transition
Temperatures Predicted Using Text Mining and Machine Learning. npj Comput. Mater..

[38] Wang B., Xu C., Zhao X., Ouyang L., Wu F., Zhao Z., Xu R., Liu K., Qu Y., Shang F., Zhang B., Wei L., Sui Z., Li W., Shi B., Qiao Y., Lin D., He C. (2024). MinerU: An
Open-Source Solution for Precise Document
Content Extraction. arXiv.

[39] Wu Z., Ramsundar B., Feinberg E. N., Gomes J., Geniesse C., Pappu A. S., Leswing K., Pande V. (2018). MoleculeNet: A Benchmark
for Molecular Machine Learning. arXiv.

[40] Delaney J. S. (2004). ESOL: Estimating
Aqueous Solubility Directly from Molecular Structure. J. Chem. Inf. Comput. Sci..

[41] Landrum, G. ; Tosco, P. ; Kelley, B. ; Rodriguez, R. ; Cosgrove, D. ; Vianello, R. ; sriniker; Gedeck, P. ; Jones, G. ; Kawashima, E. ; NadineSchneider; Nealschneider, D. ; Dalke, A. ; tadhurst-cdd; Swain, M. ; Cole, B. ; Turk, S. ; Savelev, A. ; Vaucher, A. ; Wójcikowski, M. ; Maeder, N. ; Faara, H. ; Take, I. ; Walker, R. ; Scalfani, V. F. ; Probst, D. ; Ujihara, K. ; Pahl, A. ; godin, guillaume. ; Lehtivarjo, J. Rdkit/Rdkit: 2025_09_2 (Q3 2025) Release, 2025. https://zenodo.org/records/17495409 (accessed Nov 12, 2025).

