# Supplementary material for: Machine-Learning Predictions of Photoluminescence in Molecules Exhibiting Thermally Activated Delayed Fluorescence with Implicit Experimental Validation
Source: J Chem Inf Model. 2026 May 13;66(10):5757–63. doi: 10.1021/acs.jcim.6c00425 (PMC13213825; doi:10.1021/acs.jcim.6c00425)
Supplement: Supplementary file 1 [file ci6c00425_si_001.pdf]

Supporting Information

Machine-learning Predictions of Photoluminescence in Molecules Exhibiting Thermally Activated Delayed Fluorescence with Implicit Experimental Validation

Dingyun Huang and Jacqueline M. Cole

Ray Dolby Centre, Cavendish Laboratory, Department of Physics, University of Cambridge, J. J. Thomson Avenue, Cambridge CB3 0US. U.K.

TABLE OF CONTENTS

S1 The Text-mining Workflow used to Collect TadfPL Data Records 1
S2 Statistics of TadfPL, ESOL, and Lipophilicity Datasets 3
S3 Hash Collisions in Molecular Fingerprints 4
S4 GNN Model Configuration 7
S5 Baseline Reference Models 9
References 11

S1 The Text-mining Workflow used to Collect TadfPL Data Records

The TadfPL dataset was collected by mining text about organic thermally activated delayed fluorescence (TADF) molecules from the Royal Society of Chemistry (RSC), Elsevier, and Wiley publishers, using the pipeline proposed by Huang and Cole[1]. Overall, the data-extraction process involves three stages: (1) searching and downloading relevant publications from each publisher, (2) mining text for properties and chemical names, and (3) post-processing and cleaning. The following modifications have been implemented to boost extraction precision and enhance code efficiency.

During paper retrieval, an additional requirement was set in the search query that the word "delayed" must appear in the full text of a paper with a minimum frequency of three. Keyword frequencies of common phrases in the field of TADF revealed that this can function as a simple criterion for further screening out irrelevant papers and reduce waste of computing resources.

A new workflow was also developed for mining portable document formate (PDF) files of Wiley journals so that more data could be accessed. The software tool, MinerU, was first applied to convert Wiley PDFs into markdown formatted text [2]. Markdown files follow syntax rules for formatting, such as levels of headings and equations. Text-mineable ChemDataExtractor Text objects[3,4] can be constructed following these markdown syntax hints. However, as markdown only has limited support for table structures, MinerU stores converted tables as strings in Hyper-Tex-Markup Language (HTML) format instead. Therefore, a new HTML reader was implemented in this work to correctly parse the HTML table strings from MinerU into ChemDataExtractor Table objects.[3,4]

In this work, only those photoluminescence (PL) wavelength records that have been measured in solution were extracted to ensure data consistency for modeling. Accordingly, we extracted data only from tables in the documents because table headers and footnotes allow an easy separation of PL wavelengths that have been measured in solution from electroluminescence (EL) and other PL measurements in films and devices. Thus, ChemDataExtractorTADF[1] was re-programmed so that the footnote texts were appended to the content of corresponding table cells. This modification enabled the more facile extraction of solvent names and temperature values and the better distinction of PL wavelengths from EL measurements. The table caption was also extracted along with each record, which allowed easier data cleaning in the later stages of the operational pipeline of data extraction.

Table S1. Four major types of errors occurring in the data-extraction process.

Type	Description
I	Film records and device records.
II	Duplicate or contradictory records with the same SMILES string and solvent name.
III	Incorrect solvent for Wiley entries.
IV	Data records selected by outlier detection.

During post-processing, the data format was standardized, and Simplified Molecular Input Line Entry System (SMILES) strings were computed for valid IUPAC names. The dataset was then cleaned and corrected for four major types of extraction errors identified for our extraction pipeline, as listed in **Table S1**. A record, once found incorrect, is either fully corrected and labeled as ground truth or removed from the dataset.

Type I errors include extracted records of PL or EL wavelengths measured in thin films and devices. They can be identified automatically via the filtering of data records that do not have an extracted solvent field, and their contexts that do not contain keywords about chemical solutions. Type II errors contain duplicate or contradictory records. By grouping the data entries using canonical SMILES strings, solvent names, and Document Object Identifiers (DOIs), duplicate or contradicting data can be readily observed. Type III errors involve records with incorrect solvent names that have been extracted from Wiley journals, owing to the need to employ PDF-to-text conversion. Tables in PDFs are detected and extracted using Optical Character Recognition (OCR) techniques, where a table itself, its captions, and footnotes are seen as individual floating elements on a page, and hyperlinks between table cells and footnotes cannot be extracted either. Both contributed to a high error rate in extracting solvent names and other experimental conditions that are associated with PL wavelength measurements. Finally, an unsupervised outlier detection algorithm with k-nearest neighbors was implemented. This enabled the detection of Type IV errors.

Molecules are first vectorized using RDKit fingerprints into vectors of size 8192, where each element in a fingerprint vector represents a subgraph in the molecule's skeletal formula[5]. Principal component analysis (PCA) is then performed on the fingerprint vectors, projecting them down to two-dimensions. Figure 2c from the main text of the paper illustrates a resulting 2-D scatter plot of the molecules in our dataset. Outliers are then detected through majority voting when the data-point's value deviates by at least three standard deviations from the mean values of its four nearest neighbors. The method approximates that the Euclidean distances between molecular fingerprints are positively correlated with the difference in their PL wavelengths. Despite this being a crude approximation, the method was tested to be effective on the emission-wavelengths dataset that had been extracted[6], where 113 outliers were detected with a precision of 77%.

S2 Statistics of TadfPL, ESOL, and Lipophilicity Datasets

Table S2 lists the statistics of molecules in the TadfPL, ESOL, and Lipophilicity datasets in terms of their numbers of non-hydrogen atoms.

Table S2. Descriptive statistics (mean, standard deviation, and the first and third quartile values) for the distributions of TadfPL, ESOL, and Lipophilicity datasets, in terms of the numbers of non-hydrogen atoms in each molecule.

Dataset	Mean	Std	Q1	Q3
TadfPL	58.5	25.9	42	66
ESOL	13.3	6.9	8	18
Lipophilicity	27.0	7.5	22	32

S3 Hash Collisions in Molecular Fingerprints

Generally, molecular fingerprints are subgraphs of the skeletal formula of their parent molecule. One can determine if graph isomorphisms exist between fingerprints in order to fully distinguish one fingerprint from another. However, there is no known solution to the graph isomorphism problem for general graphs within polynomial time. To overcome this issue, fingerprinting algorithms commonly use hash functions to convert molecular fingerprint graphs to numerical digits for use in downstream computational tasks[7–9]. The hash value of a fingerprint subgraph is not guaranteed to be unique and non-overlapping for distinct fingerprints. In addition, the raw hash values are often folded to fit in a vector of smaller feature sizes. Both measures can lead to hash collisions (or ``bit collisions") where two different fingerprints are mapped to the same identifier in the feature vector. When this happens, some information about the colliding features is lost, introducing noise into feature vectors. As discussed in the main manuscript, TadfPL contains much larger molecules than commonly used organic molecule benchmarking datasets; as a result, there are many more distinct fingerprints in a molecule that belong to TadfPL and the rate of hash collisions is significantly greater.

Figure S1 shows normalized histograms of the number of all fingerprints (**Figure S1a**) and the normalized numbers of unique fingerprints (**Figure S1b**) in the three datasets. These fingerprints are generated using the RDKit fingerprint algorithm. Overall, molecules in TadfPL contain a larger total number of fingerprints than those from ESOL and Lipophilicity. In contrast, the numbers of unique fingerprints in molecules from TadfPL and Lipophilicity follow a similar distribution pattern, from which we can deduce that TadfPL molecules contain many duplicate fingerprints. This can be attributed to the fact that TadfPL molecules often contain multiple fused-ring systems. Subgraph fingerprints of these chemicals usually show substantial redundancy due to repeating aromatic units within fused-ring systems and the presence of identical fused-ring systems at multiple positions in donor-acceptor (D-A) type molecules.

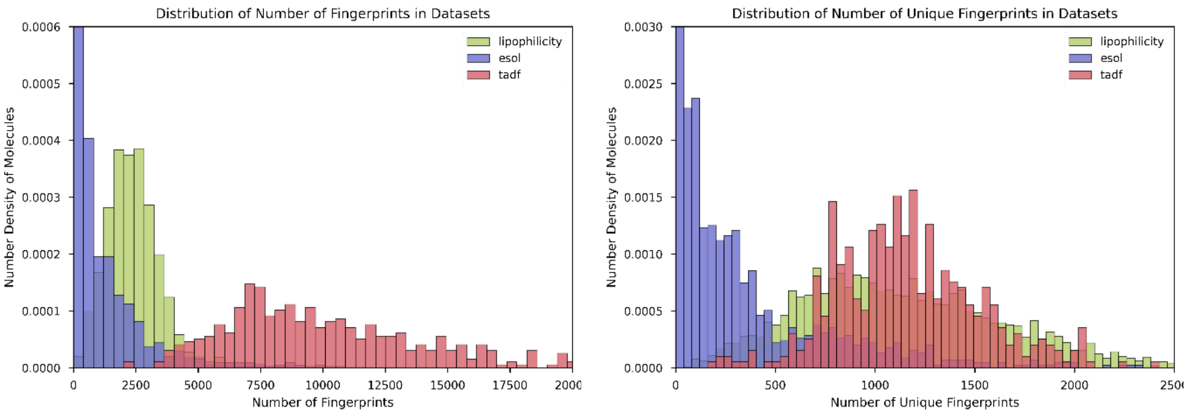

(a) **(b)**

Figure S1. Normalized histogram of **(a)** fingerprint counts and **(b)** the number of unique fingerprints in three datasets, Lipophilicity, ESOL and TadfPL. Molecules in TadfPL, on average, possess more fingerprints than those in Lipophilicity and ESOL, whereas the number of unique fingerprints in TadfPL molecules is not significantly higher than that of Lipophilicity. This observation is likely due to the presence of multiple identical moieties within TadfPL molecules, which leads to the generation of duplicate fingerprints.

Using the above generated fingerprints, the hash collision rates were calculated to be 7% for ESOL, 23% for Lipophilicity, and 23% for `TadfPL`. The algorithm used to generate the above results searched for subgraphs with one to seven edges, which are relatively small compared to the overall sizes of TADF molecules. Most of the found molecular substructures are located within a donor (D) or acceptor (A) moiety. Larger fingerprints need to be included in order to capture more long-range structural information, whereas this will boost the number of fingerprints exponentially and further raise the rate of hash collisions.

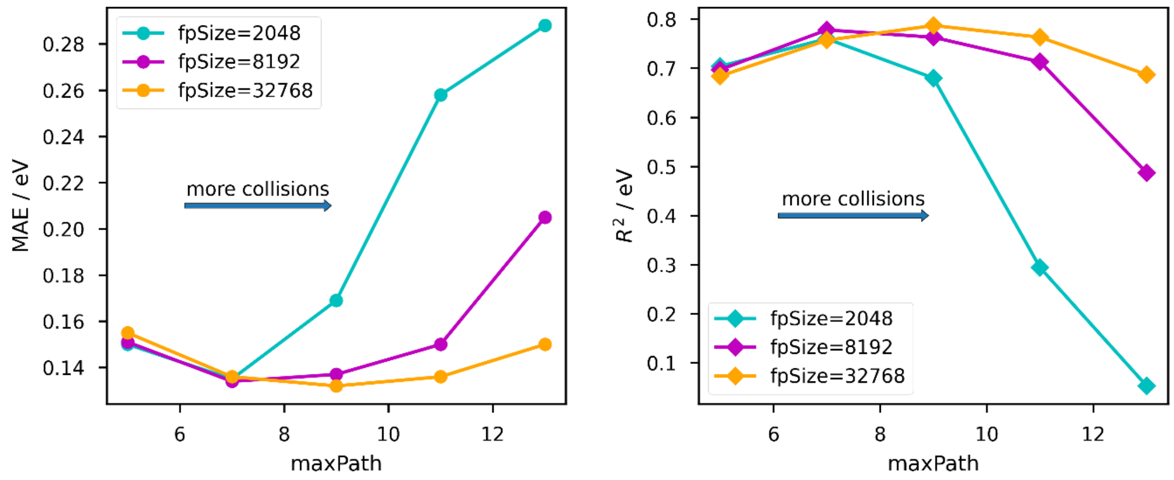

(a) **(b)**

Figure S2. The **(a)** mean absolute errors (MAEs) and **(b)** R^2s of ridge-regression models trained on RDKit branched fingerprints of `TadfPL` molecules, using various combinations of feature vector sizes and maximum subgraph sizes. Hash collision rate increases as `maxPath` of fingerprints is raised, and a degradation in prediction performance is seen with large `maxPath`. Meanwhile, the collision rate reduces as `fpSize` increases, leading to improved metrics scores from a `fpSize` of 2048 to 32,768.

Hash collisions occur when hashing subgraph fingerprints into large integers and folding them back to a finite feature vector. These collisions can potentially cause loss of information and affect regression performance. **Figure S2** illustrates the performance from trial model predictions across different feature vector lengths and maximum subgraph sizes, highlighting how variations in hash collision rates influence model performance. Specifically, ridge-regression models are trained on the RDKit branched fingerprints generated for molecules in `TadfPL`, using various combinations of maximum path lengths and feature vector size. The MAEs and R^2 scores of the models, evaluated on an out-of-sample test set, are plotted in **Figure S2a** and **Figure S2b**, respectively. The rate of hash collision is reduced when a larger feature size or a smaller maximum path length is used. For `maxPath` > 8, the regression models always perform better with a larger `fpSize`, which can be attributed to reduced hash collision rates associated with larger `fpSize`. Under the same `fpSize`s, models demonstrate the best performance when `maxPath` is set to around 7 to 9. As `maxPath` increases from a small value such as 5, the fingerprints progressively capture a larger range of structural information, and hence the regression errors are reduced. Up to a `maxPath` value of around 10, this positive effect starts to be balanced off by the negative impact brought about by large hash collision rates, which results in the shapes of MAE curves seen in **Figure S2**. Extrapolating from the

trend discovered in the exploration of hash collisions, optimal performance can be obtained around with `minPath` = 4, `maxPath` = 10, and `fpSize` = 65,536.

S4 GNN Model Configuration

The GNN model developed in this study was based on GraphSage[10]. The original implementation of GraphSage is homogeneous; the model treats all edges in graphs as the same. We updated the model architecture to be heterogeneous, so that individual treatments were given to four different bond types, namely, single, double, triple, and aromatic bond.

Each molecule is treated as a 2-D graph, where each atom is seen as a node and each bond is seen as an edge. Each atom is represented with scalar features that are stacked into a node embedding vector. Each bond is embedded as a one-hot encoded vector that covers four bond types of single, double, triple, and aromatic bonds. In the kth message passing layer of the graph module, an atom ν updates its embedding $\boldsymbol{h}_\nu^{k-1}$ according to the embeddings of neighboring covalently connected atoms $\{\boldsymbol{h}_\mathrm{u}^{\mathrm{k}-1}, u \in \mathcal{N}(\mathrm{v})\}$ via an aggregation function. In order to deal with over-smoothing, a jumping knowledge layer is used to combine a node's embeddings at each layer of the graph module into a single feature vector as its final node embedding vector[11]. Global max pooling is performed on the final embeddings of all atoms after the jumping knowledge layer to give a graph-wise feature vector, which is then propagated through the Multi-Layer Perceptron (MLP) module that outputs the predicted PL wavelength for the input molecule.

A hyperparameter sweep was conducted using Weight & Biases[12] by minimizing the prediction errors on the validation set of TadfPL with the Bayesian method[13]. Meanwhile, the total number of parameters in the GNN model is minimized when the validation error does not change significantly between configurations. All optimal hyperparameter values are listed in Table S3.

Table S3. Optimal hyperparameter combination for the heterogeneous GraphSage model developed in this study.

Hyperparameter	Values
Epoch	400
Activation Function	SiLU
Aggregation Method	Sum
Batch Size	64
MLP Dropout Rate	0.1
Hidden channels	28
Node Embedding Input Noise	0.1
Jumping Knowledge	concatenation
Learning Rate	0.005
MLP Hidden Dimension	28
Number of GNN Layers	8
Optimizer	AdamW
Scheduler	Cosine Annealing

S5 Baseline Reference Models

A Gradient Boosted Decision Trees (GBDT) model and an original GraphSage model were trained as additional baseline references for this work. The GBDT model was implemented with Scikit-Learn, and its optimal hyperparameters were given in Table S4. The optimal hyperparameters of the original GraphSage model are provided in Table S5. The optimal GraphSage model is six times as large as the HGNN introduced in this study. To enable a fairer comparison, an ablation study was conducted by training a small original GraphSage model with a size comparable to that of the HGN. The configuration of this smaller GraphSage model is summarized in Table S6.

Table S4. Optimal hyperparameter combination for the GBDT model.

Hyperparameter	Values
learning_rate	0.1
n_estimators	100
max_depth	3
min_samples_split	3
min_samples_leaf	1

Table S5. Optimal hyperparameter combination for the original GraphSage model.

Hyperparameter	Values
Epoch	400
Activation Function	SiLU
Aggregation Method	Sum
Batch Size	64
MLP Dropout Rate	0.1
Hidden channels	64
Node Embedding Input Noise	0.1
Jumping Knowledge	concatenation
Learning Rate	0.005
MLP Hidden Dimension	128
Number of GNN Layers	20
Optimizer	AdamW
Scheduler	Cosine Annealing

Table S6. Hyperparameter combination for the small GraphSage model that was configured with a size similar to that of HGNN.

Hyperparameter	Values
Epoch	400
Activation Function	SiLU
Aggregation Method	Sum
Batch Size	64
MLP Dropout Rate	0.1
Hidden channels	32
Node Embedding Input Noise	0.1
Jumping Knowledge	concatenation
Learning Rate	0.005
MLP Hidden Dimension	32
Number of GNN Layers	16
Optimizer	AdamW
Scheduler	Cosine Annealing

References

(1) Huang, D.; Cole, J. M. A Database of Thermally Activated Delayed Fluorescent Molecules Auto-Generated from Scientific Literature with ChemDataExtractor. *Sci Data* **2024**, *11* (1), 80. DOI: https://doi.org/10.1038/s41597-023-02897-3.

(2) Wang, B.; Xu, C.; Zhao, X.; Ouyang, L.; Wu, F.; Zhao, Z.; Xu, R.; Liu, K.; Qu, Y.; Shang, F.; Zhang, B.; Wei, L.; Sui, Z.; Li, W.; Shi, B.; Qiao, Y.; Lin, D.; He, C. MinerU: An Open-Source Solution for Precise Document Content Extraction. arXiv September 27, 2024 revision. http://arxiv.org/abs/2409.18839 (accessed: 2025-07-22).

(3) Swain, M. C.; Cole, J. M. ChemDataExtractor: A Toolkit for Automated Extraction of Chemical Information from the Scientific Literature. *J. Chem. Inf. Model.* **2016**, *56* (10), 1894–1904. DOI: https://doi.org/10.1021/acs.jcim.6b00207.

(4) Mavračić, J.; Court, C. J.; Isazawa, T.; Elliott, S. R.; Cole, J. M. ChemDataExtractor 2.0: Autopopulated Ontologies for Materials Science. *J. Chem. Inf. Model.* **2021**, *61* (9), 4280–4289. DOI: https://doi.org/10.1021/acs.jcim.1c00446.

(5) Landrum, G.; Tosco, P.; Kelley, B.; Rodriguez, R.; Cosgrove, D.; Vianello, R.; sriniker; Gedeck, P.; Jones, G.; Kawashima, E.; NadineSchneider; Nealschneider, D.; Dalke, A.; tadhurst-cdd; Swain, M.; Cole, B.; Turk, S.; Savelev, A.; Vaucher, A.; Wójcikowski, M.; Maeder, N.; Faara, H.; Take, I.; Walker, R.; Scalfani, V. F.; Probst, D.; Ujihara, K.; Pahl, A.; godin, guillaume; Lehtivarjo, J. Rdkit/Rdkit: 2025_09_2 (Q3 2025) Release, 2025. https://zenodo.org/records/17495409 (accessed 2025-11-12).

(6) Huang, D.; Cole, J. M. A Database of Thermally Activated Delayed Fluorescent Molecules Auto-Generated from Scientific Literature with ChemDataExtractor. *Figshare* **2023**. DOI: https://doi.org/10.6084/m9.figshare.24004182.v1.

(7) Rogers, D.; Hahn, M. Extended-Connectivity Fingerprints. *J. Chem. Inf. Model.* **2010**, *50* (5), 742–754. DOI: https://doi.org/10.1021/ci100050t.

(8) Awale, M.; Reymond, J.-L. Atom Pair 2D-Fingerprints Perceive 3D-Molecular Shape and Pharmacophores for Very Fast Virtual Screening of ZINC and GDB-17. *J. Chem. Inf. Model.* **2014**, *54* (7), 1892–1907. DOI: https://doi.org/10.1021/ci500232g.

(9) Capecchi, A.; Probst, D.; Reymond, J.-L. One Molecular Fingerprint to Rule Them All: Drugs, Biomolecules, and the Metabolome. *Journal of Cheminformatics* **2020**, *12* (1), 43. DOI: https://doi.org/10.1186/s13321-020-00445-4.

(10) Hamilton, W. L.; Ying, R.; Leskovec, J. Inductive Representation Learning on Large Graphs. arXiv September 10, 2018 revision. http://arxiv.org/abs/1706.02216 (accessed: 2025-10-24).

(11) Xu, K.; Li, C.; Tian, Y.; Sonobe, T.; Kawarabayashi, K.; Jegelka, S. Representation Learning on Graphs with Jumping Knowledge Networks. arXiv June 25, 2018 revision. http://arxiv.org/abs/1806.03536 (accessed: 2025-07-16).

(12) *Weights & Biases: The AI Developer Platform*. Weights & Biases. https://wandb.ai/site/ (accessed 2025-12-14).

(13) *Sweep configuration options*. Weights & Biases Documentation. https://docs.wandb.ai/models/sweeps/sweep-config-keys (accessed 2025-12-14).
